# Evolution of comorbidities in people living with HIV between 2004 and 2014: cross-sectional analyses from ANRS CO3 Aquitaine cohort

**DOI:** 10.1186/s12879-020-05593-4

**Published:** 2020-11-16

**Authors:** F. Bonnet, F. Le Marec, O. Leleux, Y. Gerard, D. Neau, E. Lazaro, P. Duffau, O. Caubet, M. A. Vandenhende, P. Mercie, C. Cazanave, F. Dabis

**Affiliations:** 1 CHU de Bordeaux, Service de Médecine Interne et Maladies Infectieuses, Hôpital Saint-André, 1 rue Jean Burguet, 33000 Bordeaux, France; 2 CHU de Bordeaux, COREVIH AQUITAINE, 33000 Bordeaux, France; 3 Université de Bordeaux, INSERM U1219, ISPED, 33000 Bordeaux, France; 4 CH de Dax, Service de Maladies Infectieuses, 40100 Dax, France; 5 CHU de Bordeaux, Service des maladies Infectieuses et Tropicales, 33000 Bordeaux, France; 6 CH de Libourne, Service de Maladies Infectieuses, 33500 Libourne, France

**Keywords:** HIV, Aging, Comorbidities, Cardiovascular events, Chronic kidney disease

## Abstract

**Background:**

The objective of the study was to describe the evolution of chronic non-AIDS related diseases and their risk factors, in patients living with HIV (PLHIV) in the French ANRS CO3 Aquitaine prospective cohort, observed both in 2004 and in 2014 in order to improve long-term healthcare management.

**Methods:**

The ANRS CO3 Aquitaine cohort prospectively collects epidemiological, clinical, biological and therapeutic data on PLHIV in the French Aquitaine region. Two cross sectional analyses were performed in 2004 and 2014, to investigate the patient characteristics, HIV RNA, CD4 counts and prevalence of some common comorbidities and treatment.

**Results:**

2138 PLHIV (71% male, median age 52.2 years in 2014) were identified for inclusion in the study, including participants who were registered in the cohort with at least one hospital visit recorded in both 2004 and 2014. Significant increases in the prevalence of diagnosed chronic kidney disease (CKD), bone fractures, cardiovascular events (CVE), hypertension, diabetes and dyslipidaemia, as well as an increase in treatment or prevention for these conditions (statins, clopidogrel, aspirin) were observed. It was also reflected in the increase in the proportion of patients in the “high” or “very high” risk groups of the disease risk scores for CKD, CVE and bone fracture score.

**Conclusions:**

Between 2004 and 2014, the aging PLHIV population identified in the French ANRS CO3 Aquitaine prospective cohort experienced an overall higher prevalence of non-HIV related comorbidities, including CKD and CVD. Long-term healthcare management and long-term health outcomes could be improved for PLHIV by: careful HIV management according to current recommendations with optimal selection of antiretrovirals, and early management of comorbidities through recommended lifestyle improvements and preventative measures.

## Background

Life expectancy of people living with HIV (PLHIV) has increased in the last two decades, due to improvements in patient care, including highly effective antiretroviral therapy (ART), HIV RNA suppression and CD4 cell count recovery [1, 2]. However, some analyses have shown that life expectancy in PLHIV remains lower compared to the general population. These differences, while they are more pronounced in lower income countries, are present across diverse settings (low and high income) ranging from 60% of HIV-negative life expectancy in Rwanda, to 90% in Canada [3].

Many factors have been associated with mortality, and observed decreases in life-expectancy, among PLHIV. Globally, there is evidence that women have a decreased life expectancy compared to men [3]. In Switzerland, differences in mortality were associated with educational attainment; life expectancy for PLHIV, 20 years or older, was 52.7 years (95% CI 46.4–60.1) for those with only compulsory education (16 years-old), compared to 60.0 years (95% CI 53.4–67.8) for those with higher education. Male gender, smoking, injection drug use, and low CD4 cell counts at enrolment were also independently associated with mortality in PLHIV [4].

At the same time, as the rate of mortality in PLHIV continues to decrease, the causes of death have shifted from AIDS to non-AIDS related causes, including cancers, complications of hepatitis coinfections, cardiovascular diseases, and non-AIDS infections [5]. Compared to the general population, PLHIV are more likely to develop comorbidities due to increasing age, higher exposure to risk factors, comorbidity risk associated with certain ARTs, and HIV related factors. Understanding these comorbidities is important to improve long-term healthcare management of PLHIV. The observational hospital-based prospective cohorts provide a useful tool for examining factors and long-term health outcomes within this population [6–8]. Evidence generated from these analyses could be important for policy-makers to project and plan for future needs for areas of care and associated costs [9, 10].

The objective of the present study was to describe the prevalence of chronic non-AIDS related diseases and their risk factors in aging patients included in the French ANRS CO3 Aquitaine prospective cohort, observed ten years apart (2004 and 2014).

## Methods

In the ANRS CO3 Aquitaine Cohort, HIV-1 infected patients in nine public hospitals of the Aquitaine region in Southwestern France, were followed prospectively since 1987. Details of the cohort have been described in detail previously [10]. The ANRS CO3 Aquitaine Cohort was approved by the French Commission Nationale de L’informatique et des Libertés (CNIL). Participants provided written informed consent and the study adhered to the principles of Declaration of Helsinki.

The inclusion criterion for this analysis was ≥1 hospital visit in both calendar years, 2004 and 2014. Two descriptive, cross sectional analyses were performed at the beginning (2004) and at the end (2014) of the follow-up period. During each cross-sectional period, data were collected on patient characteristics, HIV markers, prevalence of comorbidities (at least once during follow up) and treatment recorded in that year (ART and comorbidity related medication). The ANRS CO3 Aquitaine cohort prospectively collects epidemiological, clinical, biological and therapeutic data on PLHIV in the French Aquitaine region. It also records clinical events and comorbidities through a simplified version of the International Classification of Diseases 10th Revision (ICD-10). The comorbidities recorded in the present analysis were selected as the most common non HIV-related comorbidities in aging populations and were the following: cardiovascular events (CVE) (myocardial infarction, invasive coronary procedure or stroke); chronic kidney disease (CKD) (defined as eGFR< 60 mL/min/1.73 m^2^ for ≥3 months or recorded presence of an ICD-10 diagnosis code, by the physician in charge of the patient); acute kidney disease (AKD) (diagnosis of AKD (ICD-10)); bone fractures (any location; not differentiated according to high or low energy); hypertension (systolic blood pressure ≥ 140 mmHg and/or diastolic blood pressure ≥ 90 mmHg or taking antihypertensive drugs without history of cardiovascular event); dyslipidaemia (elevated total cholesterol ≥6.2 mmol/l (240 mg/dL), and/or elevated LDL-cholesterol ≥4.1 mmol/L (160 mg/dL) and/or decreased HDL-cholesterol ≤0.9 mmol/L (35 mg/dL), and/or elevated triglycerides ≥2.3 mmol/L (200 mg/dL); diabetes (diagnosis of diabetes (at least one event within ICD-10 codes), or two consecutive blood glucose results fasting or random) ≥ 7 mmol/L or taking antidiabetic drugs or insulin); obesity (Body Mass Index (BMI) ≥30 kg/m^2^); depression (diagnosis (ICD-10) or receiving specific therapy); hepatitis B virus infection (at least one HbsAg+); hepatitis C virus infection (at least one anti-HCV+ or RNA+); and cancer (non-AIDS related). FRAX (Fracture Risk Assessment) score [11], D: A: D coronary risk score [12], and D:A:D renal risk score [13] were calculated to estimate the risk of fracture, cardiovascular disease and renal disease, respectively. All comorbidity data were assessed for both time points (2004 and 2014).

To detect differences between 2004 and 2014, t-tests were used on paired quantitative data (or the nonparametric alternative - Wilcoxon test); for paired qualitative data, McNemar’s test or extended McNemar’s tests were used (or the nonparametric alternative - Exact McNemar’s test). For results, *p*-values < 0.05 were considered statistically significant. All analyses were performed with SAS 9.3 (SAS Institute, Cary, NC, USA).

## Results

In the cohort, 3289 PLHIV had at least one hospital visit registered in 2004 and 3880 in 2014;2138 PLHIV had a visit in both years and were included in this analysis.

Among the 1051 PLHIV patients seen in 2004 but not in 2014, 421 died, and 730 were lost to follow-up. Patients who died between 2004 and 2014 differed from included patients. Patients who died between 2004 and 2014 were older (mean age 48.1 vs 43.3), more likely to be male (79% vs 71%), injection drug users (37% vs 18%), with lower CD4 counts (mean 380/mm^3^ vs 496/mm^3^) and less likely to be virally suppressed (< 50 copies/mL: 42% vs 51%). Patients lost to follow-up were similar to the 2138 patients followed at two time points (data not shown). They were slightly younger and more likely to be virally suppressed (54% vs 49%).

Of the 2138 included patients, 71% were males. In 2014, 62.3% were ≥ 50 years compared to 20.3% in 2004 (Table 1), 72.0% of patients achieved CD4 counts ≥500/mm^3^ compared to 43.6% in 2004 (*p* < 0.0001) and 91.5% achieved viral suppression (< 50 copies/ml) vs 50.9% in 2004 (*p* < 0.0001) (Table 2), showing that HIV markers have markedly improved over time.

**Table 1:** Sociodemographic characteristics and risk factors, in 2004 and 2014

	2004	2014
	(*n* = 2138)	(*n* = 2138)
Male Gender, n (%)	1517 (71.0)	
Age (years), median (IQR)	42.2 (37.7;48.1)	52.2 (47.6;58.1)
Age > 50 years, n (%)	434 (20.3)	1334 (62.3)
Scholarity level, n (%)		
Primary and Secondary	28 (59.6)	
University	18 (38.3)	
Others	1 (2.1)	
BMI, median (IQR)	22.3 (20.1;24.5)	23.1 (20.7;25.9)
HIV risk factors, n (%)		
MSM	863 (40.4)	
Heterosexual	701 (32.8)	
History of IDU	394 (18.4)	
Others	180 (8.4)	

Legend: *BMI* body mass index, *IQR* inter-quartile range, *MSM* men who have sex with men

**Table 2:** HIV infection and ART treatment characteristics, in 2004 and 2014

	2004	2014	***p***-value
	***n*** = 2138	***n*** = 2138	
Time since HIV diagnosis (years), mean (SD)	10.5 (5.5)	20.5 (5.5)*	< 0.0001
CD4 count (cells/mm^3^), median (IQR)	457 (321;634)	647 (479;858)	< 0.0001
CD4 count, strata, n (%)			< 0.0001
≥ 500	879 (43.6)	1451 (72.0)	
351–500	546 (27.1)	336 (16.7)	
< 350	590 (29.3)	228 (11.3)	
Patients with viral suppression (< 50 copies/ml), n (%)	1021 (50.9)	1835 (91.5)	< 0.0001
Naïve to ART, n (%)	213 (10.0)	14 (0.7)	< 0.0001
Duration of ART (months), mean (SD)	77 (39)	190 (46)*	< 0.0001
Current ART drug class, n (%)			< 0.0001
2 NRTIs + 1 PI/r	505 (23.6)	693 (32.4)	
2 NRTIs + 1 NNRTI	467 (21.8)	690 (32.3)	
2 NRTIs + II	0 (0.0)	138 (6.5)	
Other regimens	751 (35.1)	559 (26.1)	
No treatment	415 (19.4)	58 (2.7)	
AIDS stage, n (%)	442 (20.7)	525 (24.6)*	< 0.0001

Legend: *AIDS* acquired immunodeficiency syndrome, *ART* antiretroviral therapy, *IQR* inter-quartile range, *NNRTI* Non-Nucleoside Reverse Transcriptase Inhibitors, *NRTI* Nucleoside Reverse Transcriptase Inhibitors, *PI/r* ritonavir-boosted protease inhibitor, *SD* standard-deviation

*Cumulative data between 2004 and 2014

Most comorbidities were significantly more prevalent in 2014. Hypertension and dyslipidemia were highly prevalent in HIV patients, being present in 56.3 and 50.9% of patients in 2014, respectively. While the annual prevalence of dyslipidemia decreased from 68.7% in 2004 (to 50.9% in 2014, *p* < 0.0001), and the proportion of PLHIV with LDL-cholesterol above 4.1 mmol/l (160 mg/dl) decreased from 26.1% in 2004 to 21.5% in 2014 (*p* = 0.003), in contrast, hypertension (+ 37.5%; *p* < 0.0001) and CKD (+ 14.7%; *p* < 0.0001) increased significantly during the period.

**Fig. 1:**
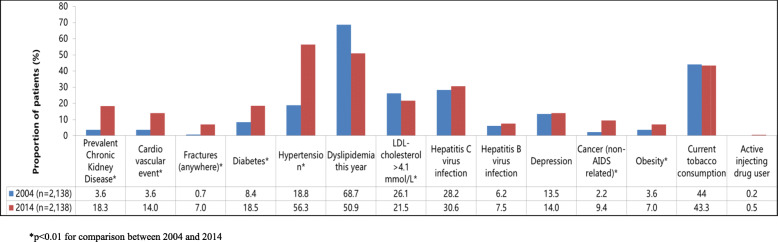
Chronic Comorbidities in 2004 and 2014, ANRS CO3 Aquitaine Cohort

The prevalence of diabetes more than doubled in 2014 compared to 2004 (18.5% vs 8.4%; p < 0.0001), while the proportion of patients with obesity increased from 3.6 to 7.0% (*p* < 0.0001). The percentage of patients with depression remained stable between the two periods (13.5 and 14.0%, *p* = 0.59) and the percentage of patients living with a history of non-AIDS cancer increased significantly from 2.2 to 9.4% (*p* < 0.0001). Current tobacco consumption remained relatively unchanged over time (44.0 and 43.3%; *p* = 0.55) (Fig. 1). Following a similar trend to the prevalence of the comorbidities, prescriptions for all concomitant medications evaluated increased significantly from 2004 to 2014 (*p* < 0.0001), except for antidepressant drugs. Blood pressure and lipid-lowering drugs showed the highest increase and were prescribed to 22.7 and 29.3% of the population analyzed in 2014, respectively. However, only 46.8% of patients with two consecutive SBP ≥ 140 mmHg and/or DBP ≥ 90 mmHg received anti-hypertensive drugs in 2014. And among the 639 patients receiving anti-hypertensive drugs, only 22.8% had their last blood pressure measured < 140/90 mmHg.

The use of statins increased from 9.2 to 24% (*p* < 0.0001) between the two periods. In 2014, patients were treated more frequently with drugs for renal conditions and prevention of cardiovascular diseases (aspirin and clopidogrel) 14.4% for renal and 10.6% (aspirin or clopidogrel) for prevention of cardiovascular diseases respectively (Table 3).

**Table 3:** Prescription of comorbidity related medication, in 2004 and 2014

	2004 (*n* = 2138)	2014 (***n*** = 2138)	*p*-value
Antidiabetics, n (%)	51 (2.4)	125 (5.8)	< 0.0001
Blood-pressure lowering treatment, n (%)	128 (6.0)	486 (22.7)	< 0.0001
Lipid-lowering treatment, n (%)	331 (15.5)	626 (29.3)	< 0.0001
Medication related to a risk of CV disease, n (%)			
Aspirin	19 (0.9)	170 (8.0)	< 0.0001
Clopidogrel	18 (0.8)	87 (4.1)	< 0.0001
Treatment associated with renal conditions, n (%)	51 (2.4)	308 (14.4)	< 0.0001
Psychotropic drugs, %	31 (1.4)	85 (4.0)	< 0.0001
Antidepressant drugs, %	197 (9.2)	215 (10.1)	0.2872

Legend: *CV* cardiovascular

The prevalence of cardiovascular and central nervous system events was approximately four times higher in 2014. History of AKD was recorded in 12.9% of patients in 2014. Approximately 10% of patients had an intermediate or high FRAX score in 2014 compared to 2.4% in 2004 (*p* < 0.0001) – results are based on data from 1884 patients with available scores in both 2004 and 2014 (observations missing data: *n* = 254; Table 4).

**Table 4:** Proportion of patients with cardiovascular, vascular central nervous system events, kidney disease and fractures, in 2004 and 2014

	2004 (***n*** = 2138)	2014 (***n*** = 2138)	***p***-value
Number of patients with cardiac, peripheral or CNS events, n (%)			< 0.0001
0	2067 (96.7)	1851 (86.6)	
1–3	67 (3.1)	234 (10.9)	
≥ 4	4 (0.2)	53 (2.5)	
Patients with history of CV events, n (%)	49 (2.3)	179 (8.4)	< 0.0001
Patients with history of CNS event, n (%)	27 (1.3)	79 (3.7)	< 0.0001
Patients with history of peripheral vascular events n (%)	10 (0.5)	127 (5.9)	< 0.0001
Chronic Kidney Disease, n (%)			
eGFR < 60	133 (7.0)	230 (12.1)	
Missing data*	237	237	
Acute kidney disease (history)	41 (1.9)	275 (12.9)	< 0.0001
FRAX score risk level, n (%)			< 0.0001
Low (< 5%)	1839 (97.6)	1701 (90.3)	
Intermediate (5–7.5%)	39 (2.1)	129 (6.8)	
High (> 7.5%)	6 (0.3)	54 (2.9)	
Missing data*	254	254	

Legend: *CV* cardiovascular, *CNS* Central Nervous System (including stroke and hemorrhage);FRAX, Fracture Risk Assessment

*Patients with missing data were not considered and results were based on the total of the remaining patients who had no missing data

**Fig. 2:**
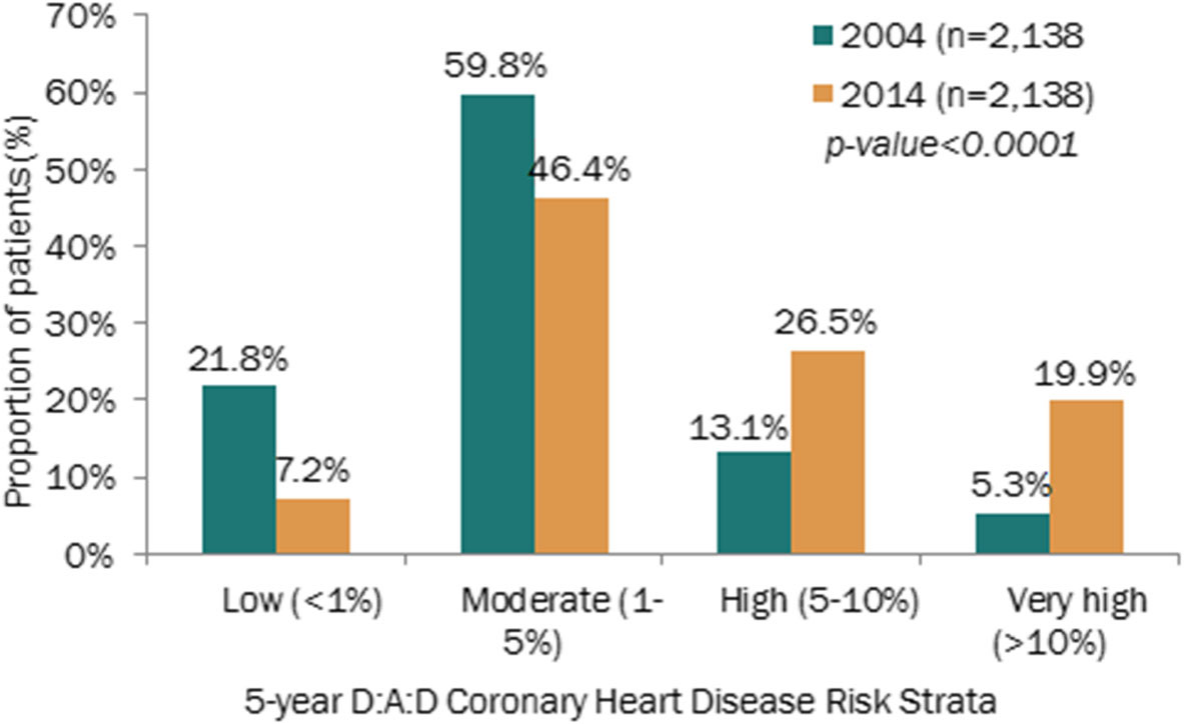
Distribution of patients by 5-year D:A:D Coronary Heart Risk Score, in 2004 and 2014

**Fig. 3:**
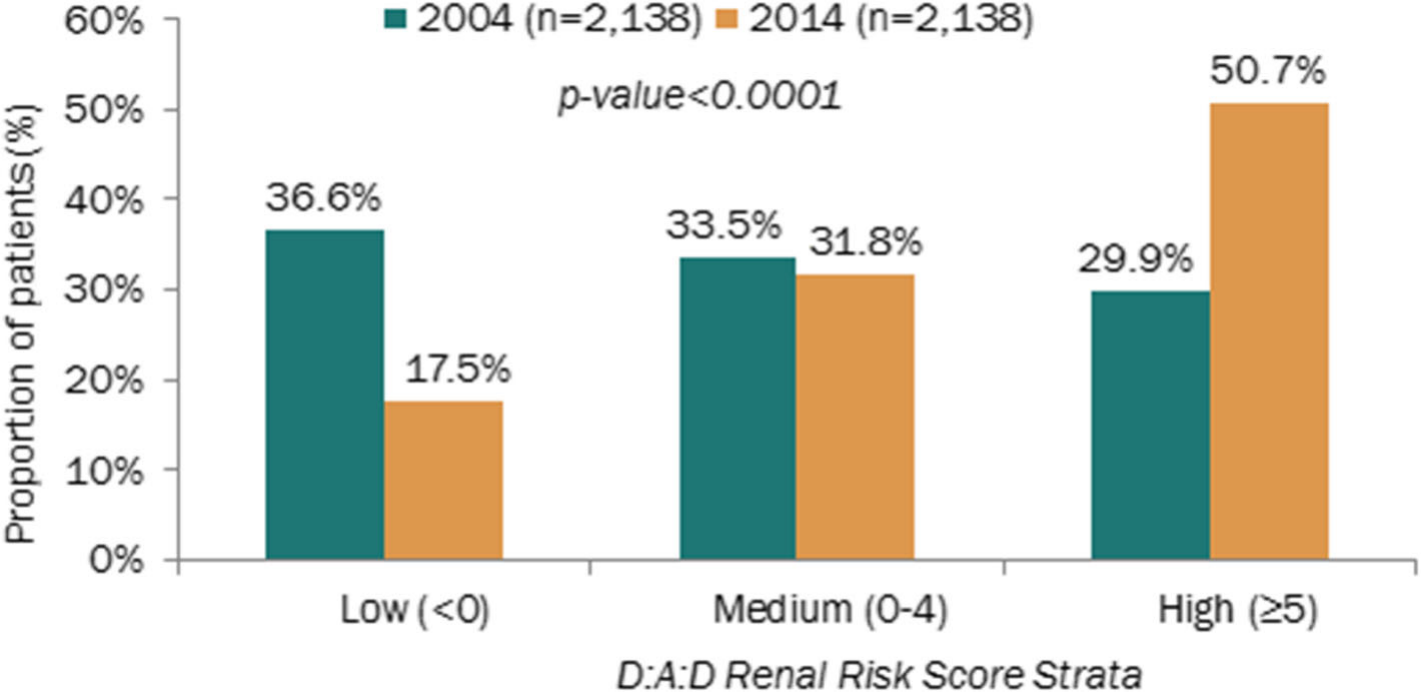
Distribution of patients by D:A:D Renal Risk Score strata, in 2004 and 2014

The proportion of patients at high or very high risk of developing coronary heart disease (CHD) over the subsequent 5 years, as measured by the D:A:D risk score, more than doubled within the 10 years of follow-up(18.4 to 46.4%;*p* < 0.001) (Fig. 2). The proportion of patients with D: A: D renal high-risk score increased from less than 30% in 2004 to over 50% in 2014 (Fig. 3).

## Discussion

In this large well-established French cohort, a significant improvement in HIV markers was observed as PLHIV aged between 2004 and 2014 - consistent with findings from prior studies. In 2014, estimates from France have shown that 84% of PLHIV have been diagnosed [14]; in Aquitaine, we estimate that approximately 90% (data not published) of PLHIV have been diagnosed and more than 95% of them are included in the ANRS CO3 Aquitaine cohort; 97.3% of the patients were treated with antiretrovirals and 91.5% had a viral load below 50 copies/mL (and only 3.4% above 1000 copies/mL), suggesting that the 90–90-90 ONUSIDA goal was within reach. Only one quarter of patients had CD4 counts that remained below 500 CD4/mm^3^ in 2014 and this proportion decreased over time. The median age of participants was 52.2 years in 2014 with 20.0% of patient being older than 60 years.

Despite the overall improvement in HIV markers over the ten-year period of this study, we also found a marked increase in the prevalence of comorbidities, risk factors associated with, and prescription of medicines to treat or to prevent, these conditions over the same period. Of the comorbidities observed, hypertension showed a particularly significant increase increasing from 18.8% in 2004 to, reaching 56.3% in 2014. These finding are similar to that in a sample comparing 540 PLHIV (median age: 52 years) to uninfected individuals from the AGEhIV Cohort study, which found the prevalence of hypertension to be 45.4% in PLHIV vs 30.5% in general population [15]. In these two studies, hypertension was the chronic comorbidity with the highest prevalence, and was present in approximately half of the patients who were 50 years and older. Recently, the French Microbreak study showed that cerebral small vessel disease (CSVD), which is a major predictor of future vascular events, cognitive impairment, frailty, and poor survival, was 60% more prevalent in PLHIV compared to the general population [16]. Age and hypertension were the main risk factors for CSVD, suggesting a possible explanation for the increased rate of neurocognitive disorders in PLHIV. It is noteworthy that in the Aquitaine clinical cohort, despite clear recommendations included in the French Guidelines from 2004, anti-hypertensive drugs were prescribed to only half of the patients with confirmed hypertension, and that among patients receiving treatment only 22.8% were adequately treated. These findings highlight that the therapeutic objectives of adequately treating blood pressure have likely not yet been achieved to prevent hypertension complications, including CSVD, in this population. Future studies should consider that optimal control of hypertension will prevent CSVD and cognitive disorders in PLHIV. In fact, previous studies have already identified that being on antihypertensive treatment was associated with a lower probability of developing cardiovascular risk [17].

The prevalence of dyslipidaemia, although decreasing between 2004 and 2014, remained at very high levels., The proportion of patients with LDL-cholesterol > 4.1 mmol/L decreased between 2004 and 2014 from 26.1 to 21.5%, possibly due to better management of dyslipidaemia, a switch of antiretrovirals (less use of boosted PIs and efavirenz) and a larger use of lipid lowering treatment (from 15.5 to 29.3% of patients; *p* < 0.0001), particularly statins.

With a prevalence of diabetes that more than doubled between 2004 and 2014 (18.5% in 2014) and a high stable prevalence of active smokers over those 10 years (43.3% in 2014), these results may explain largely the high increase of CVE between 2004 and 2014 in this cohort (14%). The prevalence of myocardial infarction and stroke was more than three times higher in 2014 than in 2004, while the prevalence of peripheral arterial disease was 10 times higher for the same period. Recent cohort studies have shown that despite higher cardiovascular disease (CVD) risk profiles for PLHIV, the incidence of CVE by age class decreased in the recent years reaching the incidence observed in the general population [18]. Once again, increased use of more lipid-friendly antiretrovirals, has increased the emphasis put on CVD risk reduction in this population. One possible explanation for these findings could be the emerging profile, in the modern ART era, of a successfully treated HIV patient. However, we have also noticed that even if 19.9% of patients were at very high risk of coronary events according to the D:A:D equation risk, only 8.0% of the total cohort were treated with aspirin, including patients in primary and secondary prevention. The prescription of statins and aspirin in PLHIV remains largely suboptimal, with less than 50% of patients requiring statins and ASA being adequately treated, based on the EACS 6:1 guideline, advising the prescription of statins for patients with CVD risk < 20% according to Framingham CHD risk stratification [19].

Tobacco cessation remains a challenge for PLHIV. Despite numerous preventive actions, the prevalence of active smokers decreased by only 0.7% between 2004 and 2014. In a US study, HIV-infected smokers aged 40 years lost > 6 years of life expectancy from smoking, possibly outweighing the loss from HIV infection itself [20]. HIV is independently associated with both smoking and not quitting smoking according to algorithm models [21]. New approaches are key necessities to improve smoking cessation levels among PLHIV [22].

CKD followed the same trend of cardiovascular comorbidities reaching 62.5% of people with creatinine clearance below 90 mL/min, including 12.1% below 60 mL/min. More than half of the patients had a high risk score of CKD according to the D:A:D renal risk score strata. Addressing risk factors such as hypertension and diabetes will continue to be of key importance in this population.

At last, the issue of depression remains of concern with a stable prevalence of approximately 14% in both 2004 and 2014. However, the prevalence of depression might be underestimated - in a sample of 400 patients evaluated for neurocognitive disorders using the CES-D depression scale, we found that 27,5% of patients had depressive symptoms in the period 2007–2009 [23].

Our study may have some limitations. Measures for blood pressure, lipids and glycaemia (fasting or not fasting) were not systematically reported and the level of abnormalities observed might be overestimated. However, we strongly believe that these conditions have remained largely unchanged between 2004 and 2014 and thus, the evolution between the two time points was probably not impacted by any overestimation. One of the criteria in capturing comorbidities, in particular hypertension, included patients taking antihypertensive drugs (ICE, β blockers). This approach could create bias in capturing the true number of patients with hypertension as these medications may be used for other indications besides hypertension. The Aquitaine Cohort collected hospital based information in the region and thus provided a good representation of PLHIV in France. However as this is limited to a specific geographical region of France, and data on socioeconomic status were not collected, the results might not be generalizable to populations with largely different profiles (e.g. dietary habits, socioeconomic status and alcohol consumption). Patients who have died before 2014 were not considered in this analysis. The aim of this approach was to ensure accurately capturing data for both time points for the purposes of comparison and patients without data for both time points were excluded regardless of the reason. Additionally, this study only included only PLHIV and did not include a control group for comparison. This is could potentially be explored in a future study using a matched control cohort to compare PLHIV to healthy controls and explore how aging could differentially affect the evolution of non-HIV comorbidities in the two populations.

As the majority of PLHIV are now over the age of 50, with age associated increasing comorbidities, the optimal HIV treatment regimen could lead to an early and effective management of these comorbidities and to a continuous improvement of PLHIV health status and quality of life. This would aim to address the effects of aging including regular monitoring and screening of major comorbidities risk modification measures, according to current recommendations, and optimal selection of ART to reduce renal and CVD risks along with balancing HIV outcomes.

In this context, effective ART options that balance HIV outcomes with a lower long-term impact on CVD, bone and renal events, would positively impact patient care and treatment. Adequate control of risk factors associated with these comorbidities, and counselling on cessation of smoking along with aiming for other lifestyle improvements, would be highly beneficial for PLHIV.

### Aquitaine cohort (France) composition

**Coordination:** F. Bonnet, F. Dabis. Scientific committee: F. Bonnet, S. Bouchet, D. Breilh, G. Chêne, F. Dabis, M. Dupon, H. Fleury, V. Gaborieau, D. Lacoste, D. Malvy, P. Mercié, P. Morlat, D. Neau, I. Pellegrin, JL. Pellegrin, S. Reigadas, S. Tchamgoué.

**Epidemiology and Methodology:** G. Chêne, F. Dabis, C. Fagard, S. Lawson-Ayayi, L. Richert, R. Thiébaut, L. Wittkop.

**Infectious Diseases and Internal Medicine:** K. André, F. Bonnet, N. Bernard, L. Caunègre, C. Cazanave, I. Chossat, C. Courtault, FA. Dauchy, S. De Witte, D. Dondia, M. Dupon, P. Duffau, H. Dutronc, S. Farbos, I. Faure, M. Foissac, V. Gaborieau, Y. Gerard, C. Greib, M. Hessamfar-Joseph, Y. Imbert, D. Lacoste, P. Lataste, E. Lazaro, D. Malvy, J. Marie, M. Mechain, P. Mercié, E. Monlun, P. Morlat, D. Neau, A. Ochoa, JL. Pellegrin, M. Pillot-Debelleix, T. Pistone, I. Raymond, MC. Receveur, P. Rispal, L. Sorin, S. Tchamgoué, C. Valette, MA. Vandenhende, MO. Vareil, JF. Viallard, H. Wille, G. Wirth. Immunology: JF. Moreau, I. Pellegrin.

**Virology:** H. Fleury, ME. Lafon, S. Reigadas, P. Trimoulet.

**Pharmacology:** S. Bouchet, D. Breilh, F. Haramburu, G. Miremont-Salamé.

**Data collection, Project Management and Statistical Analyses:** MJ. Blaizeau, I. Crespel, M. Decoin, S. Delveaux, F. Diarra, C. D’Ivernois, C. Hanappier, D. Lacoste, S. Lawson-Ayayi, O. Leleux, F. Le Marec, E. Lenaud, J. Mourali, E. Pernot, A. Pougetoux, B. Uwamaliya-Nziyumvira, R. Nawabzad. IT department and eCRF development: V. Conte, G. Palmer, V. Sapparrart, D. Touchard.

## Data Availability

All data generated or analysed during this study are included in this published article [and its supplementary information files].

## Abbreviations

AKD: Acute kidney disease
ART: Antiretroviral therapy
BMI: Body mass index
CHD: Coronary heart disease
CKD: Chronic kidney disease
CSVD: Cerebral small vessel disease
FRAX: Fracture Risk Assessment
HCV: Cardiovascular events
HIV: Human Immunodeficiency Virus
PLHIV: Patients living with HIV
